# Medicinal Value of *Camellia sinensis* Bioactive Components: Focus on Theaflavins and Tea Polysaccharides

**DOI:** 10.3390/ijms27156771

**Published:** 2026-07-29

**Authors:** Xueyan Zhao, Mostafa Sagharyan, Shirin Mohammadbagherlou, Sudarshanee Geekiyanage, Mo-Xian Chen, Litang Lu

**Affiliations:** 1 College of Life Sciences, The Key Laboratory of Plant Resources Conservation and Germplasm Innovation in Mountainous Region (Ministry of Education), Guizhou University, Guiyang 550025, China; zhhaoxy@outlook.com; 2 Center for Research and Development of Fine Chemicals, Guizhou University, Guiyang 550025, China; mostafasaghar@gmail.com (M.S.); m.bagherlou.sh@gmail.com (S.M.); 3 Department of Agricultural Biology, Faculty of Agriculture, University of Ruhuna, Mapalana, Matara 81100, Sri Lanka; sudarshanee@agri.ruh.ac.lk

**Keywords:** *Camellia sinensis*, metabolic pathways, tea polysaccharides, theaflavins

## Abstract

Tea, derived from the leaves of *Camellia sinensis*, is a non-alcoholic beverage rich in pharmacologically active bioactive components. This plant contains many bioactive components, such as xanthine alkaloids, tea polyphenols, L-theanine, theaflavins, tea polysaccharides, and volatile oils. Evidence indicates that these bioactive constituents contribute to various physiological benefits, including body weight regulation, anti-fatigue properties, hypoglycemic action, antitumor and neuroprotective potentials, improvement of vascular function, and attenuation of airway-induced organ obstruction. Up to now, research has systematically confirmed and summarized the functions of xanthine alkaloids, tea polyphenols, and L-theanine. However, much less attention has been given to theaflavins and tea polysaccharides. This review therefore focuses on how theaflavins and tea polysaccharides work in the human body and what they do, as well as how these two types of compounds are metabolized in tea plants. Overall, this work provides a new perspective on the study of bioactive components in tea and builds a useful foundation for a deeper understanding of how these components act in the human body.

## 1. Introduction

Tea (*Camellia sinensis*) has a long history of medicinal and dietary use in Asian countries such as China, Japan, India, and Thailand, dating back as far as several thousand years [1]. As one of the most popular and least expensive beverages in the world (second only to water), tea not only promotes general health but also exhibits therapeutic effects against various diseases [2]. Tea has been consumed by people from different cultures and different regions. Tea has not only become a daily beverage in many regions, but also serves, to some extent, as a kind of “health product” for maintaining the body at home.

The bioactive components of tea play important roles in a form of chemical warfare and environmental adaptation [3,4]. The bioactive components of tea plants mainly include catechins, tea polysaccharides, L-theanine, caffeine, theaflavins, and volatile oils [5].

Each component has its own unique chemical structure, function, and flavor, and together they demonstrate a broad range of biological activities, including anti-cancer potential, neuroprotective effects, antioxidant activity, and anti-inflammatory properties [6].

In recent years, the improvement of analytical technologies including high-performance liquid chromatography, mass spectrometry and nuclear magnetic resonance, alongside the progress of molecular biology, has promoted in-depth research on tea bioactive components [7]. Among them, the functional components such as catechins, L-theanine, and caffeine have been extensively studied, and their mechanisms of action in the human body are relatively well understood. Specifically, catechins are known for their strong antioxidant and anticancer activities, L-theanine has been shown to promote relaxation and improve cognitive function, and caffeine acts as a central nervous system stimulant [8,9]. Caffeine is an Xanthine alkaloid, a natural stimulant found in tea.

Caffeine competitively binds to A_1 and A_2 adenosine receptors in the brain. By blocking adenosine (which signals fatigue), it increases the release of neurotransmitters like dopamine and norepinephrine, enhancing alertness and cognitive function [10,11]. At higher concentrations, it inhibits phosphodiesterase inhibition, preventing the breakdown of cyclic AMP (cAMP), which prolongs epinephrine.

Theaflavins are a group of benzotropolone compounds formed during black tea fermentation through the oxidative polymerization of catechins catalyzed by polyphenol oxidase [12]. They mainly include theaflavin, theaflavin-3-gallate, theaflavin-3′-gallate, and theaflavin-3,3′-digallate. Tea polysaccharides, on the other hand, are acidic heteropolysaccharides often bound to proteins, composed primarily of glucose, galactose, arabinose, and xylose [13]. Although previous reviews have systematically summarized the bioactivities and future development prospects of bioactive compounds from *Camellia sinensis* [14], theaflavins which are predominantly generated during tea fermentation have not been clearly and specifically addressed in these works. As for tea polysaccharides, while reviews have described their beneficial effects on human health and discussed their pharmaceutical development potential [14], they have generally lacked in-depth mechanistic analysis and failed to provide a systematic classification of these polysaccharides. Consequently, compared with other well-studied active substances such as catechins and caffeine, systematic research on theaflavins and tea polysaccharides remains insufficient, and the knowledge gap regarding their pharmacological mechanisms, metabolic pathways, and structure–activity relationships persists. The pharmacological functions and metabolic pathways of these two types of components in the human body, as well as their synthesis and degradation mechanisms within tea plants, have not been extensively studied and still require further investigation.

This review focuses on the pharmacological effects of theaflavins (anti-inflammatory, anticancer, antioxidant, etc.) and the various activities of tea polysaccharides (antioxidant and hepatoprotective effects, antitumor activity, immunomodulation, gut microbiota regulation, regulation of glucose and lipid metabolism, etc.). It also discusses the current status of pharmaceutical development of tea bioactive components, and summarizes the synthesis and degradation pathways of theaflavins and tea polysaccharides and summarized their chemical structures, as shown in Table A1. in tea plants, with emphasis on key enzymes and regulatory steps. The goal is to provide a reference for basic research and applied development of tea bioactive components, and to facilitate their potential use as functional food ingredients.

## 2. Biological Activities of Bioactive Components from the Tea Plant in the Human Body

### 2.1. Current Status of the Medicinal Functions of Catechins, Alkaloids, and Theanine

Catechins, mainly flavan-type polyphenols with epigallocatechin gallate (EGCG) as the representative compound [15]—caffeine, a xanthine alkaloid that acts as the principal stimulant in tea [16], and L-theanine, a distinctive non-protein amino acid linked to the umami taste and neuroactive properties of tea [17]. These three classes represent the most thoroughly investigated bioactive components in tea leaves. A wealth of evidence supports the biological activities of these compounds, ranging from anticancer [18] and neuroprotection [19] to antidepressant [20], asthma relief [21], hepatoprotection [22], and weight management [22]. Given that comprehensive reviews on these subjects are already available, we will not cover the underlying mechanisms or therapeutic assessments in detail here.

### 2.2. Pharmacological Functions of Theaflavins

**Figure 1:**
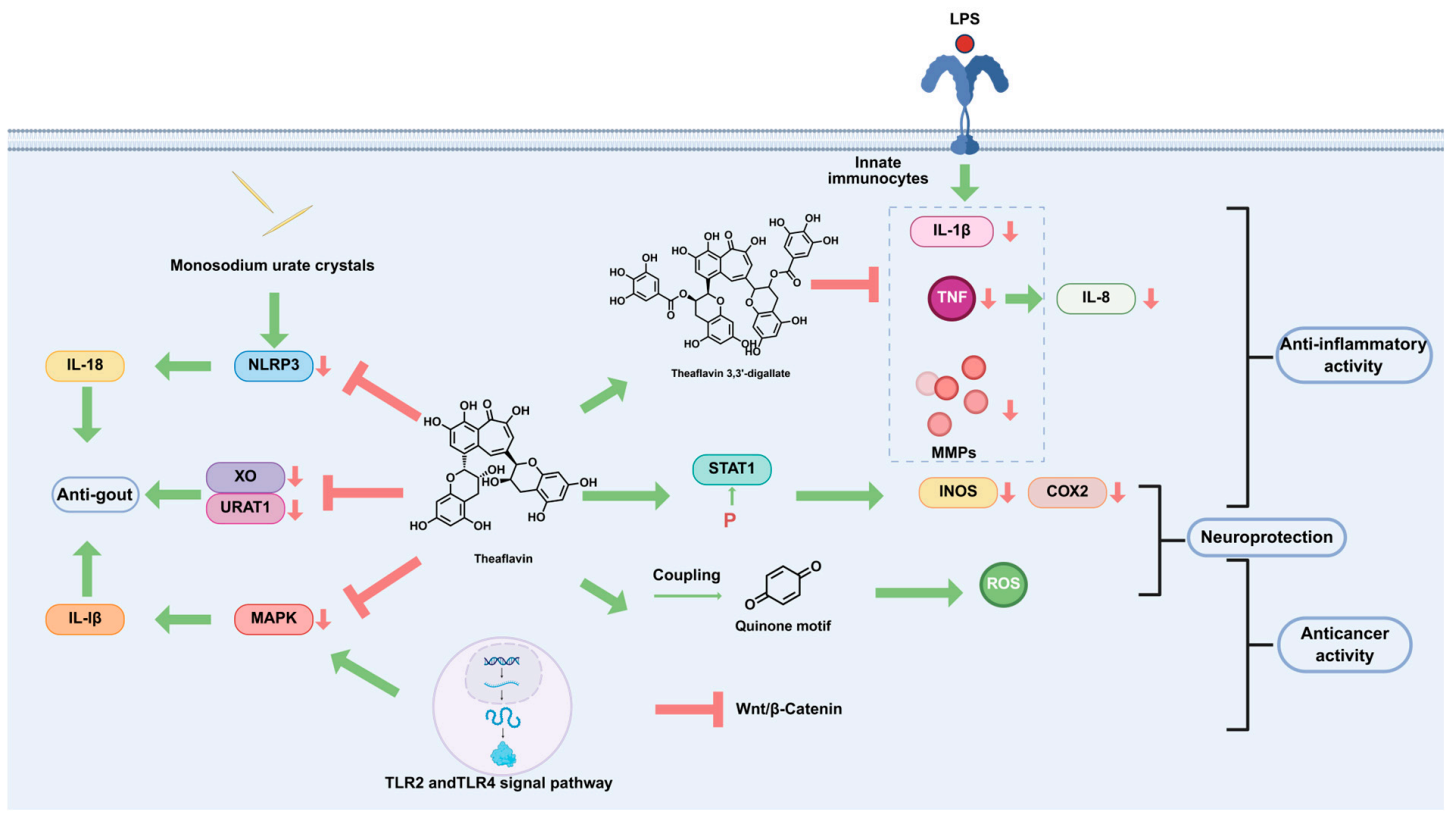
Diagram of some mechanisms through which theaflavins exert health benefits in the human body. Theaflavins modulate NLRP3 inflammasome, MAPK, and TLR2/4 signaling pathways, inhibit xanthine oxidase (XO) and URAT1, and interfere with the Wnt/β-catenin pathway, thereby exhibiting anti-inflammatory, anti-gout, urate-lowering, anticancer, and neuroprotective effects. In the figure, red arrows denote inhibition, and green arrows denote promotion.

Theaflavins are a class of benzotropolone compounds. Theaflavins are produced during the fermentation stage of black tea through the oxidative polymerization of catechins mediated by polyphenol oxidase [23]. Due to the various biological characteristics of theaflavins, there have been many related studies and reports (Table A2). Previous studies have demonstrated that theaflavins possess certain functions in anti-inflammation, anti-tumor, neuroprotection, and anti-gout effects (Figure 1).

(1) Anti-inflammatory activity

Inflammation is a basic defense mechanism coordinated by the immune system, and it can protect the body from infection and tissue damage [24]. In addition to the above-mentioned functions, it also plays an important role in maintaining tissue homeostasis. However, if inflammation persists chronically in the human body, it can also be harmful to human health [25].

Lipopolysaccharides (LPS) are fundamental glycolipids that make up the outer leaflet of the outer membrane in nearly all Gram-negative bacteria. LPS can activate innate immune cells such as macrophages. After stimulation, these cells can release free radicals, matrix metalloproteinases (MMPs), interleukin-1β (IL-1β), tumor necrosis factor (TNF), and other inflammation-related mediators, thereby triggering an inflammatory cascade [26,27]. Theaflavins interfere with this pathway at multiple steps, as shown in Figure 1. Among them, theaflavin-3,3′-digallate, a key metabolite, has been reported to exert anti-inflammatory effects by downregulating IL-1β and TNF-α expression in RAW 264.7 macrophages and PMA-primed U937 cells [28], which helps alleviate LPS-induced acute lung injury in mice. In another study, theaflavin treatment reduced TNF-α, IL-1β, and several MMPs (MMP-3, MMP-8, and MMP-9) in *Porphyromonas gingivalis*, suggesting an additional anti-inflammatory mechanism [29]. In addition, theaflavins have been found to block TNF-induced IL-8 gene transcription in A549 cells, curtailing IL-8 release [30]. In addition, theaflavins can inhibit some pro-oxidative enzymes related to inflammatory regulation, such as cyclooxygenase-2 (COX-2) and inducible nitric oxide synthase (iNOS) [31]. Theaflavins promote anti-inflammatory responses and, to some extent, enhance antioxidant defense through the above pathways (Figure 1).

(2) Anticancer activity

Cancer has remained a major global health burden over the past several decades [32], leading to sustained research into its causes, pathological characteristics, and therapeutic approaches [33]. Previous studies have shown that theaflavins are able to scavenge free radicals and inhibit the proliferation of human colon cancer SW620 and SW480 cells, suggesting their potential role in cancer prevention and treatment [34]. In addition, when conjugated with specific carriers, the quinone groups present in the structure of theaflavins can promote the production of reactive oxygen species and induce mitochondrial depolarization, thereby producing antitumor activity [35]. It has been reported that theaflavins can also control the expression of β-catenin, an important downstream factor in the Wnt signaling pathway, thereby affecting this pathway which is frequently abnormally activated in tumors, and thus expressing their antitumor activity [36]. Overall, these findings confirm that theaflavins act on cancer through the regulation of multiple mechanisms and the influence on multiple signaling pathways. However, before applying them to clinical practice, the current results are still not completely reassuring, and more studies are still needed (Figure 1).

(3) Neuroprotection

Previous studies have confirmed the mechanisms related to the pathology of neurodegenerative disorders (NDDs) [37,38]. Among these mechanisms, oxidative stress is considered to be the main “culprit” causing diseases [39] such as Alzheimer’s disease (AD), Parkinson’s disease (PD), and amyotrophic lateral sclerosis (ALS) [40]. As previously described, theaflavins further reduce pro-oxidative enzyme activity by inhibiting Signal Transducer and Activator of Transcription 1 (STAT1) phosphorylation, ultimately exerting anti-inflammatory effects. Moreover, some quinone structural units in the theaflavin molecule are conjugated, thereby binding to carriers and inducing the production of reactive oxygen species (ROS), ultimately achieving anticancer effects [41]. Notably, the diminished activity of pro-oxidative enzymes also contributes to an augmented antioxidant defense capacity within the organism, providing a potential mechanistic basis for the neuroprotective actions of theaflavins. In addition, a moderate increase in reactive oxygen species (ROS) levels can produce an activation signal that triggers the nuclear factor E2-related factor 2 (Nrf2) signaling cascade. After entering the nucleus, Nrf2 can promote the transcription of antioxidant response elements (ARE), thereby upregulating the expression of various endogenous antioxidant enzymes, such as superoxide dismutase (SOD) [42]. In summary, theaflavins can enhance cellular resistance to oxidative stress, thereby achieving the protection of nerves (Figure 1).

(4) Anti-gout

Gout is essentially a crystal-induced arthropathy caused by purine metabolic imbalance in the human body. Blood urate concentrations of gout sufferers can surpass the saturation threshold of monosodium urate within joint cavities [43]. Under such supersaturated conditions, urate crystals separate out in joints and adjacent tissues. These crystalline deposits then induce local non-bacterial inflammation, which manifests as typical acute gout attacks. In clinical treatment, gout is known for its severe and acute characteristics, and this pain usually peaks at night, which can severely affect the daily functioning of gout patients [44].

Theaflavins can intervene in gout through multiple aspects. Theaflavins can significantly reduce the gene and protein expression of URAT1 (the main renal transporter responsible for urate reabsorption), while also inhibiting the catalytic function of xanthine oxidase (XO) [45]. This effect can promote the reduction in urate production while also enhancing the renal excretion function of the human body, thereby achieving a decrease in uric acid levels under the joint efforts of these two aspects. In addition, theaflavins can also interfere with the TLR2- and TLR4-mediated signaling pathways by blocking the phosphorylation-dependent activation of p38 MAPK (MAPK14), thereby inhibiting the maturation and release of IL-1β [45,46]. Other studies have reported that the NLRP3 inflammasome assembly and caspase-1 activation induced by monosodium urate crystals are both inhibited by theaflavins, which in turn inhibits the synthesis of IL-18 in the body and alleviates the inflammatory cascade response under NLRP3 signaling, thereby suppressing gout [47]. Collectively, through their coordinated modulation of urate metabolism and multiple pro-inflammatory pathways, theaflavins hold appreciable promise as a candidate for further exploration in gout management (Figure 1).

### 2.3. Pharmacological Functions of Tea Polysaccharides

**Figure 2:**
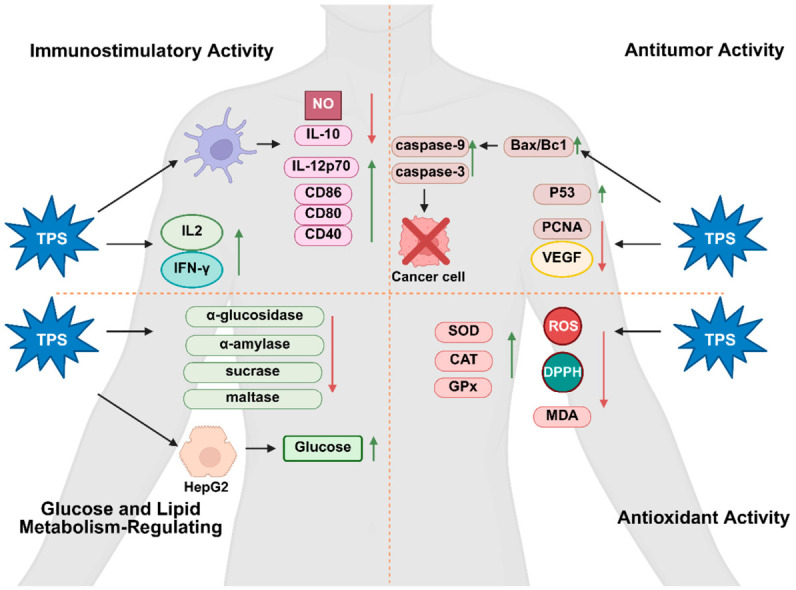
Diagram of some mechanisms through which tea polysaccharide exerts health benefits in the human body. Tea polysaccharide exerts antioxidant, antitumor, immunostimulatory, and metabolic-regulating effects via immunomodulation, apoptosis induction, angiogenesis inhibition, ROS scavenging, and digestive enzyme inhibition.

Polysaccharides are high-molecular-weight polymers composed of more than ten monosaccharide units linked by glycosidic bonds, and the formation of glycosidic bonds depends on condensation reactions (reactions that occur between the hemiacetal (or hemiketal) group of one sugar moiety and the hydroxyl group of another sugar moiety) [48]. Tea polysaccharides (TPS) are a class of polysaccharides extracted from tea plants, mainly present in the leaves, flowers, and seed coats of the plant [49]. Although bioactive components of the tea plant such as polyphenols and L-theanine have been extensively investigated, tea polysaccharides (TPS), also belonging to this group, have attracted comparatively limited research interest (Table A3). Upon intake, tea polysaccharides produce a range of beneficial effects on the human body (Figure 2).

(1) Antioxidant Activity

Tea polysaccharides have been reported to show antioxidant activity. In animal models, tea polysaccharides can markedly reduce the level of malondialdehyde (MDA), an end product of lipid peroxidation, and increase the activities of endogenous antioxidant enzymes, including superoxide dismutase (SOD), catalase (CAT), and glutathione peroxidase (GPx) [50]. These results indicate that the in vivo antioxidant effect of tea polysaccharides is at least partly related to the activation of the body’s endogenous antioxidant defense system. Through this process, tea polysaccharides may help scavenge free radicals, reduce oxidative stress-induced injury, and improve overall antioxidant capacity. TFP-1 is a high-molecular-weight, structurally defined, water-soluble polysaccharide isolated from the flowers of *Camellia sinensis*. Because of its antioxidant activity, it has received attention as a possible way to improve the value-added use of tea plant by-products. Current evidence shows that TFP-1 produces antioxidant effects by scavenging reactive oxygen species (ROS) in a dose-dependent manner [51]. The DPPH scavenging assay is commonly used to evaluate the in vitro antioxidant activity of tea polysaccharides (TPS). TPS activity differs depending on source, molecular weight, and processing method. Fractionated green tea TPS components (TPS1–TPS3) showed stronger activity than crude TPS, among which TPS1 was the most active [52]. Black tea TPS showed the highest DPPH scavenging rate (61.7%), followed by green tea (47.9%) and oolong tea (23%) [53]. Crude TPS from four tea varieties showed scavenging capacity comparable to that of ascorbic acid at 200–400 μg/mL, although their activity was weaker at lower concentrations [54]. These results support the use of DPPH activity as an in vitro indicator of the antioxidant potential of TPS. In vivo, MDA is often used as a biomarker of lipid peroxidation. In exercised animals, crude TPS administration reduced MDA levels in plasma, liver, and heart after 30 days, while also increasing antioxidant enzyme activities. TPS fractions also decreased gastric MDA levels in cancer models and improved immune parameters [55]. Taken together, TPS appears to exert antioxidant effects in vivo by inhibiting lipid peroxidation and reducing MDA production (Figure 2).

(2) Antitumor Activity

Many research groups focus on the anti-cancer capacity of tea polysaccharides. Experiments using green tea selenium-enriched polysaccharides (Se-GTPs) prove these compounds slow the growth of human MCF-7 breast tumor cells [56]. Researchers further explored the underlying molecular pathways: Se-GTPs raise the Bax/Bcl-2 ratio to switch on caspase-3 and caspase-9, and this cascade reaction induces tumor cell death. Data also reveal elevated p53 protein levels alongside cell cycle arrest, meaning the p53 signaling cascade partially mediates the anti-tumor function of tea polysaccharides (Figure 2). Another animal experiment with H22 tumor-bearing mice records lowered VEGF and PCNA levels in tumor tissue, offering extra proof for the anti-tumor value of tea polysaccharides [57].

(3) Immunostimulatory Activity

Immune regulation is one of the most well-documented biofunctions of tea polysaccharides. Huang’s team treated mouse dendritic cells with green tea polysaccharides and detected a higher expression of costimulatory markers CD86, CD80 and CD40. The treated cells secreted more IL-12p70, yet generated less NO and IL-10. Such shifts mark the functional activation of dendritic cells, which explains how tea polysaccharides adjust immune responses [58]. Han and co-workers carried out separate animal trials with tea flower polysaccharides. Medium and high dosages successfully lifted IL-2 and IFN-γ concentrations in mouse blood [59]. Such results imply tea polysaccharides reshape circulating cytokine levels to adjust the systemic immune status (Figure 2).

(4) Glucose- and Lipid Metabolism-Regulating Activity

Plenty of research confirms tea polysaccharides can balance blood sugar and lipid levels. These compounds block the activity of α-glucosidase, and stronger inhibition occurs in polysaccharide fractions rich in neutral sugars [60]. Du’s team extracted water-soluble polysaccharides from Pu-erh tea and tested its hypoglycemic effect on cell lines and animal subjects. At appropriate concentrations, the extract boosted glucose absorption in HepG2 cells and restrained the activity of intestinal sucrase, maltase and pancreatic amylase (Figure 2). All these results support the metabolic regulatory effects of tea polysaccharides [61].

(5) Anticoagulant, antibacterial, antifatigue, and skincare activities

Apart from the functions mentioned above, existing papers also record anticoagulant, bacteriostatic, fatigue-alleviating and skin-care effects of tea polysaccharides [62]. However, relevant research remains limited. Few studies clarify the exact molecular pathways behind these activities, and existing experimental data cannot fully elaborate their action mechanisms [63,64,65].

## 3. Current Status of Pharmaceutical Development of Tea Plant Bioactive Components

Lots of preclinical tests have confirmed that theaflavins and tea polysaccharides (TPS) possess multiple pharmacological functions. Even so, low bioavailability stops these two substances from being developed into clinical medicines [66]. Theaflavins can barely be absorbed through intestines, which creates big obstacles for oral delivery. Three main reasons account for this problem: they break down under different pH conditions, membrane transporters pump them out of cells, and they get metabolized quickly after entering the liver. Following ingestion, only trace amounts of theaflavins reach systemic circulation [66].

To overcome these bioavailability barriers, considerable efforts have been directed toward nanoformulation strategies. Zein-stabilized theaflavin nanoparticles prepared by antisolvent precipitation achieved an encapsulation efficiency of 84.89% and a loading capacity of 74.11%, with the nanocomplex maintaining over 70% encapsulation efficiency after 30 days of storage [66]. Chitosan-caseinophosphopeptide nanocomplexes have been shown to significantly enhance the intestinal permeability of theaflavin-3,3′-digallate using the Caco-2 monolayer model [67]. Genipin-crosslinked chitosan–polypeptide nanocomplexes further improved PH stability and enhanced the in vitro intestinal permeability of theaflavin-3,3′-digallate [68]. Iron oxide chitosan nanoparticles improved theaflavin bioavailability by approximately 3.5-fold in vivo [69]. Nanoliposome encapsulation has also been explored as a strategy to improve theaflavin bioavailability [45].

For tea polysaccharides, pharmaceutical development has followed a different trajectory. TPS possess excellent biocompatibility and good biodegradability, making them attractive biomaterials for drug delivery and food industry applications [70]. However, their high molecular weight and structural complexity have restricted purification and limited bioactivity [70]. Selenium nanoparticles stabilized and functionalized by green tea and Pu-Erh tea polysaccharides have been developed, demonstrating the excellent potential of tea polysaccharides in nanoparticle stabilization and functionalization [71]. High-internal-phase emulsions stabilized by alkali-extracted green tea polysaccharide conjugates have been investigated for curcumin delivery [72]. Green tea polysaccharide conjugates combined with bovine serum albumin have shown synergistic effects in improving emulsification ability [73].

All in all, the development of theaflavins and tea polysaccharides (TPS) as pharmaceuticals is still at an early stage. For theaflavins, the primary obstacle remains how to overcome their poor bioavailability. For TPS, the major focus has shifted toward drug delivery, emulsification, and functional food applications. Clinical translation, especially for theaflavins, will still need more systematic and practical toxicological studies in humans.

## 4. Biosynthesis and Metabolic Transformations of Tea Macromolecules

### 4.1. Synthesis and Degradation of Theaflavins

#### 4.1.1. Biosynthetic Pathway and Subsequent Oxidative Reactions of Theaflavins

**Figure 3:**
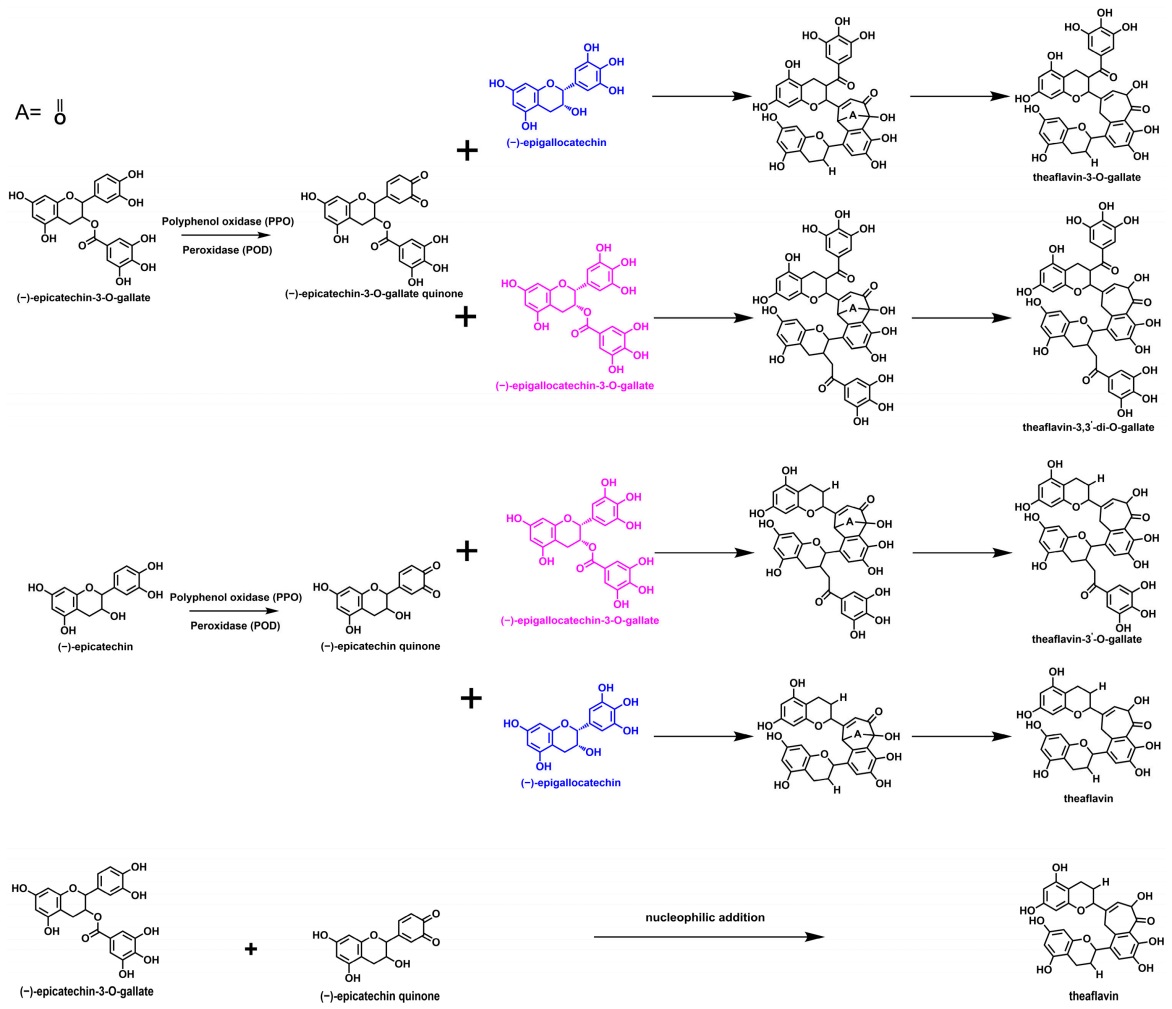
Biosynthetic pathway of theaflavins. Blue and purple represent the same compounds, and the upper pathway is the classical route. The upper route proceeds via Michael addition, carbonyl addition, three-membered ring intermediate formation, and oxidative decarboxylation, while the lower route involves nucleophilic addition of galloylated catechins.

Theaflavins are red benzotropolone pigments produced during black tea fermentation, and they account for approximately 2–6% of the dry weight of brewed black tea solids. They are formed from parent catechins, including epicatechin (EC), epigallocatechin (EGC), epicatechin gallate (ECG), and epigallocatechin gallate (EGCG), through oxidative coupling reactions catalyzed by polyphenol oxidase (PPO) or peroxidase (POD) in fresh green tea leaves [74,75,76]. In the classical biosynthetic pathway of theaflavin, EC is first oxidized by PPO or POD to form EC quinone, which then oxidizes EGC and generates EGC quinone. A Michael addition reaction between EGC quinone and EC quinone then takes place, followed by carbonyl addition and the formation of a three-membered ring intermediate. Subsequent oxidation and decarboxylation produce the benzotropolone skeleton that is characteristic of theaflavin [77]. Nakatsuka et al. (2005) later proposed another biosynthetic route, in which EGC directly attacks EC quinone through nucleophilic addition at the initial stage, offering a different explanation for the enzymatic formation of theaflavins [78]. Theaflavins do not act as the metabolic endpoint of black tea manufacturing; rather, they are structural intermediates in a continuous oxidative cascade. If fermentation is prolonged or excessive heat occurs during drying, coupled chemical auto-oxidation and continued enzymatic activity polymerize monomeric theaflavins into heterogeneous, high-molecular-weight brownish-red polymers known as thearubigins [79,80]. Over-oxidation further degrades thearubigins into dark brown, water-soluble sedimented polymers called theabrownins, a progression that fundamentally alters the sensory profile by diminishing the briskness and brightness of the tea infusion (Figure 3) [81].

#### 4.1.2. Upstream Transcriptional Regulation in the Tea Plant

While the physical formation of the aflavins occurs post-harvest during processing, the chemical capacity of the leaf to synthesize TFs is strictly governed pre-harvest at the genetic level. R2R3-MYB transcription factors (such as CsMYB1, CsMYB5b, and CsMYB75) are critical regulators of the phenylpropanoid and flavonoid biosynthetic pathways in *Camellia sinensis* [82,83]. These MYB factors form a regulatory complex (often interacting as an MBW complex with Basic Helix-Loop-Helix [bHLH] and WD40 proteins) to directly bind to the promoters of structural genes like phenylalanine ammonia-lyase (*PAL*), chalcone synthase (*CHS*), and leucoanthocyanidin reductase (*LAR*). The up-regulation of these MYB factors increases the accumulation of the specific starter catechins (EC, EGC, ECG, EGCG) required for TF synthesis. Conversely, specific repressor MYBs belonging to subgroup 4 (like CsMYB4a) can actively down-regulate the phenylpropanoid and shikimate pathways, decreasing catechin synthesis under shifting environmental or developmental conditions [84].

The abundance of substrate catechins and the activity of endogenous polyphenol oxidase/peroxidase are regulated by complex gene networks controlled by key transcription factors (TFs): Environmental stresses, including mechanical wounding and plucking, trigger transcriptional reprogramming that modulates secondary metabolite biosynthesis [85]. The expression of genes encoding polyphenol oxidases (*CsPPOs*) and peroxidases (*CsPODs*) is similarly modulated by transcriptional regulators reacting to mechanical wounding, plucking stress, and phytohormones (such as jasmonic acid and salicylic acid). For instance, the R2R3-MYB transcription factor CsMYB59 acts as a direct transcriptional activator that binds to and up-regulates the promoter of *CsPPO1*. High transcriptional expression of *CsPPO* genes in the healthy leaf, induced by native defense mechanisms or developmental cues, ensures a massive reservoir of latent enzymes localized within the chloroplasts, primed to mix with vacuolar catechins during the mechanical rolling phase of black tea processing [86].

### 4.2. Synthesis and Degradation of Tea Polysaccharides

Tea polysaccharides (TPS) comprise a complex arrangement of various monosaccharide units, possessing distinct structural features that dictate their physicochemical and biological properties. Most TPS molecules exist as complex glycoconjugates characterized by one or more carbohydrate chains covalently bound to a specific polypeptide backbone via either N- or O-glycosidic linkages, thereby forming a highly heterogeneous macromolecular matrix [87]. The carbohydrate component within this matrix is typically composed of diverse monosaccharides, predominantly rhamnose, arabinose, xylose, mannose, galactose, glucose, galacturonic acid, and glucuronic acid [88]. Consequently, the classification of TPS into distinct structural categories stems from inherent variations in total molecular weight, precise monosaccharide molar ratios, and the localized tissue matrix from which they are isolated, such as tender flushes, coarser leaves, or mature tea flowers [88]. While the precise genetic blueprint orchestrating the synthesis of this complex tea glycoconjugate matrix remains to be fully elucidated, the baseline sugar nucleotide biosynthetic pathway serves as the universal origin point for these complex macromolecules.

**Figure 4:**
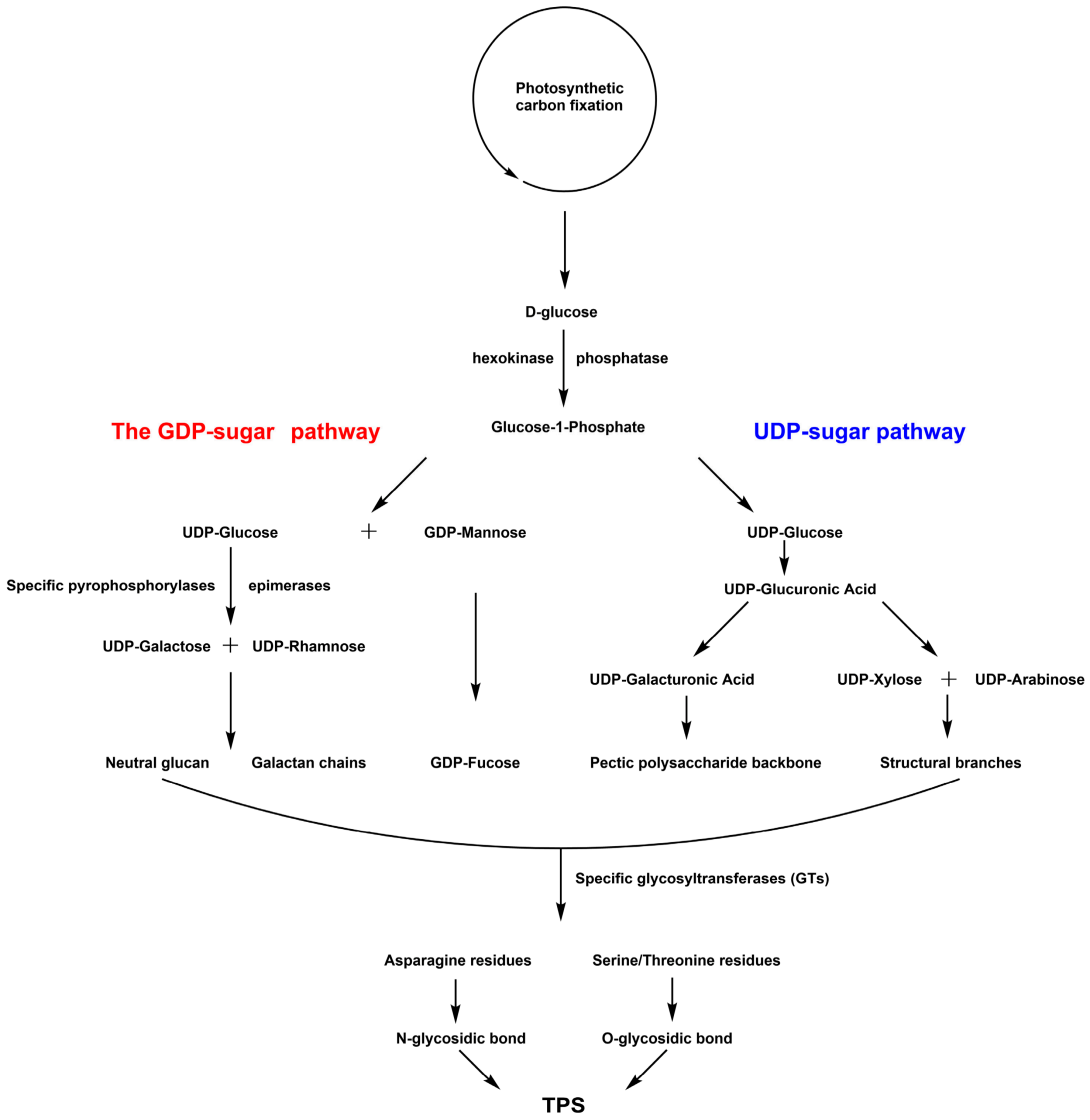
Biosynthetic pathway of TPS. Photosynthesis-derived D-glucose is converted to glucose-1-phosphate, which enters the UDP-sugar and GDP-sugar pathways to generate activated nucleotide sugars. These are subsequently transferred by glycosyltransferases to polypeptide backbones via N- or O-glycosidic bonds, yielding the TPS glycoprotein matrix.

The initialization of TPS biosynthesis begins with free D-glucose derived from photosynthetic carbon fixation via the Calvin cycle. This initial carbon substrate undergoes phosphorylation and subsequent enzymatic conversions mediated by hexokinases and phosphatases to form glucose-1-phosphate (Glucose-1-P), which acts as the precursor pool for downstream sugar nucleotide assembly. This pool splits into two primary pathways to generate activated donor molecules essential for polymer construction: the uridine diphosphate (UDP)-sugar pathway and the guanosine diphosphate (GDP)-sugar pathway, yielding UDP-glucose and GDP-mannose, respectively. Downstream of GDP-mannose, conversions lead to the formation of GDP-fucose. Concurrently, UDP-glucose serves as a pivotal metabolic branch point; it is converted via specific pyrophosphorylases and epimerases into UDP-galactose (UDP-Gal) and UDP-rhamnose (UDP-Rha), which function as the primary building blocks for neutral glucan and galactan chains. Alternatively, UDP-glucose is oxidized into UDP-glucuronic acid (UDP-GlcA), which is further converted into UDP-galacturonic acid (UDP-GalA) to form the foundational pectic polysaccharide backbone. Downstream decarboxylation pathways also convert UDP-GlcA into UDP-xylose (UDP-Xyl) and UDP-arabinose (UDP-Ara) to supply the structural branches required for hemicellulose-like fractions (Figure 4).

Following the generation of these activated sugar nucleotides, the processes of glycosylation and protein backbone linking are mediated by specialized glycosyltransferases (GTs) localized within the endoplasmic reticulum (ER) and the Golgi apparatus. These GTs catalyze the precise transfer of activated sugar moieties onto the target polypeptide backbone, establishing highly stable N-glycosidic bonds at asparagine residues or O-glycosidic bonds at serine or threonine residues, which ultimately yields the biologically active, therapeutic TPS glycoprotein matrix. This review summarizes these polysaccharide fractions, and their detailed information is provided in Table 1.

This complex synthesis of cell wall-associated polysaccharides and intracellular glycoproteins is tightly regulated by an intricate network of transcriptional regulators, wherein the R2R3-MYB transcription factor family serves as a core coordinator [96,97]. Within this network, transcription factors such as the MYB46 and MYB83 orthologs function as master molecular switches governing cell wall matrix formation [97]. When up-regulated by developmental signals or exogenous stimuli such as root-colonizing plant growth-promoting bacteria (PGPB). These specific MYB factors bind directly to the promoter regions of downstream structural genes, thereby activating cellulose synthase complexes (CESAs) and up-regulating the targeted glycosyltransferases responsible for weaving the structural xylan and pectin chains of the final tea polysaccharide complex.

## 5. Limitations and Future Development on the Utilization of Theaflavins and Tea Polysaccharides

Although theaflavins and tea polysaccharides have shown great pharmacological promise in preclinical studies, their clinical translation faces major hurdles. Since the limitations of these two compounds are essentially different, future research should pursue distinct directions.

The core bottleneck for theaflavins lies in their extremely low in vivo bioavailability, a problem caused by multiple interrelated factors [66]. Theaflavins exhibit very poor solubility in water [98], undergo degradation under different pH conditions in the gastrointestinal tract, are pumped out of cells by membrane transporters such as P-glycoprotein [99], and are rapidly metabolized upon entering the liver. These obstacles collectively result in only trace amounts of theaflavins reaching systemic circulation after oral administration [99]. In addition, the high production cost of theaflavins and the fact that their mechanisms of action in the human body remain largely unclear further hamper their clinical application, while the scale-up from laboratory to industrial production still faces significant technical challenges [100,101]. Future research should focus on several key areas, including the further optimization of nano-delivery systems to develop formulations with greater translational potential, the conduct of systematic pharmacokinetic and toxicological studies in humans to establish safe dosage ranges, the in-depth elucidation of the molecular mechanisms through which theaflavins exert their effects in the human body, and the full consideration of actual bioavailability levels when attributing in vivo biological activities to these compounds [45,98].

The challenges faced by tea polysaccharides are fundamentally different from those of theaflavins. The primary issues with tea polysaccharides lie in their extreme structural complexity and excessively high molecular weight [102]. Tea polysaccharides are not single compounds but complex heteropolysaccharides composed of more than ten monosaccharides linked by various glycosidic bonds, with molecular weights ranging from several kDa to thousands of kDa. Moreover, tea polysaccharides tend to form hyperbranched structures and aggregates in solution, often existing as glycoprotein complexes and forming natural complexes with tea polyphenols, proteins, and other components, which further complicates structural elucidation [87,103,104]. These structural characteristics make extraction and purification extremely difficult; although traditional hot water extraction is low-cost, it tends to cause glycosidic bond cleavage and thermal degradation, while strong acid or strong alkaline conditions may alter the polysaccharide conformation and lead to loss of bioactivity [105]. Because the precise molecular structure of tea polysaccharides has not yet been fully elucidated, the structure–activity relationship between their bioactivity and structure remains difficult to establish, severely limiting their application [106].

Future research should focus on the continuous and in-depth elucidation of the precise molecular structure of tea polysaccharides to establish a clear structure–activity relationship [106]. Extraction and purification processes should be optimized, with exploration of newer extraction technologies to reduce costs, improve efficiency, and obtain polysaccharide fractions with more defined structures and more stable bioactivity [107]. Meanwhile, the ongoing development of tea polysaccharide-based applications in biofilm products (such as wound dressings), drug delivery carriers, and functional foods (such as emulsifiers, edible films, and gut microbiota regulators) will further brighten the future prospects of tea polysaccharides [106].

Both theaflavins and tea polysaccharides face the common challenge of insufficient clinical research in their development as pharmaceuticals. The vast majority of current studies remain at the in vitro and animal levels, and clinical studies involving human subjects are extremely scarce. Furthermore, the safety, absorption mechanisms, and release behavior of nanoformulations themselves still require systematic evaluation. Although nano-delivery strategies hold great promise, large-scale, long-term, and well-designed clinical intervention studies are still needed to verify their efficacy and safety before they can be truly translated into clinical applications.

## 6. Conclusions and Perspectives

This review consolidates the pharmacological properties, formulation development, and biosynthetic pathways of two relatively underexplored tea bioactive components—theaflavins and tea polysaccharides. Despite growing evidence supporting their therapeutic potential, clinical research on these compounds remains insufficient.

Theaflavins exhibit anti-inflammatory, anticancer, neuroprotective, and anti-gout activities through mechanisms involving modulation of inflammatory mediators, apoptosis, Wnt/β-catenin signaling, Nrf2-driven antioxidant responses, and NLRP3 inflammasome function. Tea polysaccharides demonstrate antioxidant, antitumor, immunostimulatory, hypoglycemic, and lipid-lowering effects via enhancement of endogenous antioxidant systems, immune regulation, gut microbiota modulation, and carbohydrate digestion inhibition. Notably, the Nrf2/ARE axis appears to be a common mechanistic hub, with theaflavins acting through direct Keap1 modification and tea polysaccharides via AMPK-mediated Nrf2 activation.

Despite encouraging preclinical findings, clinical translation faces significant hurdles. For theaflavins, poor bioavailability, driven by low solubility, gastrointestinal degradation, and rapid clearance, remains the primary obstacle. Various nano-delivery systems have been explored, but clinical evidence is still lacking. Safety concerns, including cytotoxicity at high concentrations, mild hepatotoxicity in animal models, and rare allergic reactions, also warrant further toxicological evaluation. For tea polysaccharides, structural heterogeneity and high molecular weight complicate purification and compromise bioactivity; batch-to-batch variability and potential immunogenicity further highlight the need for standardized preparation protocols.

Future research should prioritize the development of novel delivery carriers for theaflavins, especially those enabling targeted accumulation and sustained drug release [108]; for tea polysaccharides, efforts should focus on elucidating structure–activity relationships to develop polysaccharide-based therapeutic products [109]. Additional studies are needed to clarify their gut microbiota-modulating effects, and rigorous human clinical trials are required to validate efficacy and safety. Integration of pharmaceutical sciences, molecular pharmacology, and clinical research will be essential to advance the therapeutic application of these two tea-derived components.

## Figures and Tables

**Table 1:** Classification of tea polysaccharides (TPS) under different categories.

Classification Basis	Category	Abbreviation/Name	Source/Characteristics	Example	Reference
By plant part	Tea leaf polysaccharide	TLPS	From tea leaves	Rha, Ara, Gal, Glu, Xyl, GalA, Man, Rib	[89]
	Tea flower polysaccharide	TFPS/TFP	From tea flowers	Rha, Ara, Gal, Glu, Xyl, GalA, Man	
	Tea seed polysaccharide	TSPS	From tea seeds	Rha, Ara, Gal, Glu, Xyl, GalA	
By tea type (fermentation degree)	Green tea polysaccharide	GTPS	Unfermented	Gal, Glu	[90]
	Oolong tea polysaccharide	OTPS	Semi-fermented	TTPS, FTPS, DTPS	[91]
	Black tea polysaccharide	BTPS	Fully fermented	Arabinogalactan	[92]
	Pu-erh tea polysaccharide	PTPS/WEPT	Post-fermented	Lac, Ara, Man, Glc, XylRha	[93]
By molecular weight	High molecular weight	HMW-TPS	Ultrafiltration fractions	>100 kD	[52]
	Medium molecular weight	MMW-TPS	Ultrafiltration fractions	10 kD–100 kD	
	Low molecular weight	LMW-TPS	Ultrafiltration fractions	<10 kD	
By purity	Crude polysaccharides	Crude TPS	Molecular weight cut-off	Contains polyphenols, proteins	[52]
	Purified fractions	Purified fractions	Molecular weight cut-off	TF-1(mannose-rich), TF-2 (xylose-rich), TF-3 (xylose-rich)	[52,55]
By chemical modification	Selenium-containing tea polysaccharides	Se-GTPs	Selenized green tea polysaccharides	The same source type of polysaccharide	[56]
	Protein-bound polysaccharides	TPS-protein conjugates	Covalently bound with protein		[94]
By solubility	Water-soluble polysaccharides	Water-soluble TPS	Hot water	More protein and uric acid; a lower content of neutral sugars	[95]
	Alkali-soluble polysaccharides	Alkali-soluble TPS	Alkali water	Less protein and uric acid; a higher content of neutral sugars	

Note: Lactose (Lac), arabinose (Ara), mannose (Man), glucose (Glc), xylose (Xyl), rhamnose (Rha), galactose (Gal), galacturonic acid (GalA), ribose (Rib).

## Data Availability

No new data were created or analyzed in this study. Data sharing is not applicable to this article.

## Appendix A

**Table A1:** Chemical structures of the major compounds mentioned in this article.

Compound Name	Chemical Structure	Reference
(−)-Epicatechin (EC)		[77]
(−)-Epicatechin-3-O-gallate (ECG)		
(−)-Epigallocatechin (EGC)		
(−)-Epigallocatechin-3-O-gallate (EGCG)		
(−)-Epicatechin quinone		
(−)-Epicatechin-3-O-gallate quinone		
Theaflavin (TF)		
Theaflavin-3-O-gallate (TF-3-G)		
Theaflavin-3′-O-gallate (TF-3′-G)		
Theaflavin-3,3′-di-O-gallate (TFDG)		
Caffeine		[110]
L-Theanine		[111]
UDP-Glucose UDP-Galactose UDP-Rhamnose UDP-Xylose UDP-Arabinose	Their chemical structures are almost identical, differing only in the spatial orientation of the hydroxyl group (-OH) on the carbon atom (C).	[112,113]
DP-Mannose GDP-Fucose	Their chemical structures are almost identical, differing only in the spatial orientation of the hydroxyl group (-OH) on the carbon atom (C).	[114,115]
DP-Glucuronic Acid UDP-Galacturonic Acid	Their chemical structures are almost identical, differing only in the spatial orientation of the hydroxyl group (-OH) on the carbon atom (C).	[113]

**Table A2:** Summary of representative experimental studies on theaflavin.

Experimental Type	Research Content	Experimental Model/Methods	Key Findings	Reference
In Vitro	Autophagy induction in Ehrlich’s Ascites Carcinoma (EAC) cells	EAC cell culture; Trypan-blue exclusion, MTT assay, Monodansylcadaverine staining, Western blotting	Theaflavins caused ~8% EAC cell death in vitro, significantly reduced metabolic activity, and induced conspicuous vacuolization (autophagosomes) confirmed by LC3II augmentation; no significant apoptosis observed up to 12 h	[116]
In Vivo	Renal ischemia/reperfusion injury (RIRI) protection	C57BL/6J male mice; 45 min ischemia + 24 h reperfusion; theaflavin intervention	Theaflavin exerted protective effects against RIRI by inhibiting apoptosis and oxidative stress via regulation of the p53/GPx-1 pathway	[117]
	Antiviral activity against Zika virus	ZIKV-infected mouse model; TF2b treatment	TF2b improved survival rate of infected mice; viral load was higher in spleen and blood, followed by liver, epididymis, and testis	[117]
	Autophagy induction in EAC tumor-bearing mice	EAC-bearing mice; oral theaflavins (10 mg/kg b.w.) every alternate day, 27 doses total	Oral theaflavins restricted excessive body-weight increase, reduced tumor volume, increased survivability; caused ~30% EAC cell death in vivo	[116]
	Inhibition of B16F10 melanoma tumor growth	B16F10 melanoma mouse model; theaflavin at 40–120 mg/kg	Theaflavin significantly inhibited B16F10 tumor size; exhibited higher antitumor efficacy than cisplatin	[118]
In Silico	Theaflavin as mTOR inhibitor for cancer therapy	Molecular docking against mTOR (PDB ID: 4FA6); pharmacokinetic properties, drug-likeness, MM-GBSA binding energy	Theaflavin identified as a strong mTOR inhibitor and apoptosis inducer through in silico approaches	[119]
	LXR-β agonist screening of four theaflavin monomers	Molecular docking, free energy calculations, molecular dynamics simulations (TF1, TF2A, TF2B, TF3 against human LXR-β)	TF2B (theaflavin-3′-gallate) identified as a putative LXR-β agonist	[120]
	Inhibition mechanism of theaflavins on MMP-2	Enzyme inhibition kinetics, multispectral analysis, molecular docking, molecular dynamics simulations	Four theaflavins (TF1, TF2A, TF2B, TF3) competitively and reversibly inhibited MMP-2 via noncovalent interactions (hydrogen bonds, hydrophobic interactions); inhibitory potency: TF1 > TF2B > TF2A > TF3	[121]
	Anti-cariogenic properties against Streptococcus mutans	Molecular docking; ligands prepared and optimized using Avogadro-1.2	Theaflavins exhibited significant binding affinities to various virulence-associated proteins of *S. mutans*	[122]
	Inhibitory mechanisms of galloylated theaflavins on α-glucosidase	Molecular docking and molecular dynamics simulations	Molecular docking and MD simulations predicted binding modes between theaflavins and α-glucosidase; galloyl groups enhanced binding affinity	[123]
Clinical Trial	Effect of theaflavin on oral bacterial flora	Randomized double-blind study in healthy volunteers (JPRN-UMIN000020049)	Completed trial evaluating the effect of theaflavin on oral bacterial flora in healthy volunteers	[124]

**Table A3:** Summary of representative experimental studies on tea polysaccharides.

Experimental Type	Research Content	Experimental Model/Methods	Key Findings	Reference
In Vitro	Lipid-lowering effects of Fuzhuan brick tea polysaccharides	Oleic acid-induced HepG2 cells	FTP3 alleviated lipid accumulation, activated AMPK, and downregulated SREBP-1C and FAS expression	[125]
	Gut microbiota and SCFA regulation	In vitro fecal microbiota fermentation	Selectively enriched SCFA-producing gut microbiota	[126]
	Bioactivities of Liupao tea polysaccharides	In vitro antioxidant, hypoglycemic, and hypolipidemic assays	TPS-5 (Mw 48.289 kDa) exhibited ferric ion reducing power, digestive enzyme inhibitory activity, and bile salt-binding capacity	[127]
In Vivo	Probiotic activity of An tea polysaccharides (ATPSs)	C57BL/6J mice; ATPSs administration; gut microbiota analysis	ATPSs promoted the levels of Lactobacillus and Bifidobacterium in vivo, confirming their prebiotic potential	[128]
	Anti-obesity mechanism of Shuiman tea polysaccharide STPS-3-1	HFD-induced male C57BL/6J mice (6 weeks, 18–20 g); 12-week STPS-3-1 intervention	STPS-3-1 improved blood glucose and lipid levels, mitigated metabolic disorders, reduced inflammation, reshaped gut microbiota, enriched Bacteroides vulgatus LWZ 240601 which degraded STPS-3-1 via PULs and regulated bile acid metabolism through FXR/SHP pathway	[129]
	Tea flower polysaccharide (TFPS) against metabolic syndrome	HFD-induced mice; TFPS at 400 and 800 mg/kg·BW/d oral administration	TFPS significantly reduced body weight, fat accumulation, plasma TC, TG, LDL-C, and pro-inflammatory cytokines (TNF-α, IL-6, IL-1β), while increasing IL-10; modulated gut microbiota diversity, affecting 11 genera including Lactobacillus and Lactococcus	[130]
	Tea polysaccharide TPS3A against renal tubular ectopic lipid deposition	HFD-exposed ApoE−/− mice; palmitic acid-stimulated HK-2 cells	TPS3A improved kidney function, slowed tubulointerstitial fibrosis, suppressed lipogenesis (down-regulated SREBP-1 and FAS), enhanced lipolysis (up-regulated HSL and ATGL) via AMPK-SIRT1-FoxO1 signaling axis	[131]
	Effects of TPS on gut microbiota and physiological health	Mice; high-dose (TPH) and low-dose (TPL) TPS groups	TPS increased beneficial probiotics (Bacteroidota, Muribaculum), decreased detrimental bacteria (Prevotella_copri, Desulfovibrionia), lowered AST/ALT ratio and creatine kinase, elevated serum amylase and glucose in TPH mice; no adverse effects on liver or intestinal integrity	[132]
In Silico	Qingzhuan tea polysaccharide-F^−^ binding behavior	Molecular structure analysis; binding behavior study	Investigated the binding behavior of purified Qingzhuan tea polysaccharides (pTPS) with fluoride ions and their regulatory role in digestion and metabolism	[133]
Clinical Trial	-	-	-	-
